# Study on the Hydration Kinetics Characteristics of Low-Calcium Cementitious Materials Based on Alkali-Activated CWM

**DOI:** 10.3390/ma19102027

**Published:** 2026-05-13

**Authors:** Shengbo Zhou, Gengfei Li, Jian Wang, Kai Zhang, Shengjie Liu

**Affiliations:** 1 School of Civil Engineering and Architecture, Suqian University, Suqian 223800, China; 2 School of Civil Engineering, Hohai University, Nanjing 210024, China

**Keywords:** construction waste micro-powder, alkali activation, hydration kinetics, synergistic effect

## Abstract

This study systematically investigated the alkali activation behavior of construction waste micro-powder (CWM) to develop a low-carbon, high-performance cementitious material. The activator formulation was optimized, the hydration thermodynamics were analyzed, and a kinetics model was constructed to reveal the reaction mechanism. The composite activator (sodium silicate and Portland cement) exhibited a significant synergistic effect, outperforming single activators. The optimal ratio was determined: 40% CWM, 60% Portland cement, and 8% water glass (modulus 1.0), which balances the system’s alkalinity and silicate modulus. Thermogravimetric analysis revealed a notable net weight gain at 3 days, indicating an ongoing secondary hydration reaction. By 7 days, the main hydration was complete, accompanied by microstructural densification, which confirmed the efficiency of the composite activator. A key contribution was the successful application of the Krstulović–Dabić (KD) model to quantify the hydration mechanism. The hydration process evolved sequentially through nucleation and growth (NG, dominant before 0.05~0.15 h), phase boundary reaction (I), and diffusion (D). The period of 0.21–50 h was governed by both I and D, after which D became the sole rate-limiting step. The model yielded the rate constants (*K*_NG_, *K*_I_, *K*_D_), Avrami exponent (n), and transition points (α1, α2), providing a kinetic explanation for the ‘early strength and rapid hardening’ characteristic. In conclusion, this work establishes a material design framework guided by activator optimization, supported by thermodynamics, and explained by kinetics. The KD model proves to be a powerful tool for deciphering the hydration behavior of alkali-activated CWM, offering theoretical guidance for developing sustainable cementitious materials with controllable performance.

## 1. Introduction

Rapid urbanization continues to drive a significant increase in construction waste. China’s annual construction waste production surpassed 3 billion tons in 2023, accounting for over 40% of total urban waste, and is expected to reach 4 billion tons by 2025 [1]. Among this waste stream, construction waste muck—rich in silicate and aluminosilicate minerals—holds significant potential for high-value utilization in building materials beyond simple backfilling [2]. Driven by the national “dual-carbon” goals, research into valorizing such waste into construction materials has gained paramount importance. Through alkali-activation (geopolymerization), construction waste micro-powder (abbreviated as CWM, the product obtained by drying and grinding the construction waste muck) has been successfully transformed into novel low-calcium cementitious systems [3].

Cement production is accompanied by significant CO_2_ emissions, with over 70% of anthropogenic CO_2_ originating from fossil fuel combustion. To promote green development of the cement industry, measures such as improving energy efficiency, using supplementary cementitious materials, and developing low-carbon cementitious materials have been implemented [4]. Low-calcium cementitious materials based on CWM can reduce emissions, save resources, and lower costs, aligning with sustainable development requirements.

In the field of solid waste resource utilization, alkali activation technology has become a research hotspot due to its advantage of “treating waste with waste” [5]. It uses strong alkalis or alkali salts to activate the active silica–alumina components in solid waste to generate cementitious products, and has been applied to steel slag, fly ash, and other solid wastes [6,7,8,9]. For example, alkali-activated steel slag-slag concrete can replace 30–50% of Portland cement, reduce CO_2_ emissions by more than 40%, and achieve a compressive strength of 40–60 MPa [10].

Low-calcium cementitious materials based on CWM contain 20–35% of active SiO_2_ and Al_2_O_3_. By adjusting the alkali equivalent and modulus, the 28-day compressive strength can reach 25–35 MPa [11]. However, the hydration kinetics of this material remain poorly understood: fluctuations in muck composition make reaction rate control difficult, the evolution of intermediate products is unclear, and existing models are difficult to adapt, affecting mix proportion optimization.

Although the Krstulović–Dabić (KD) model has been widely applied to characterize the hydration kinetics of alkali-activated slag, fly ash, and hybrid cementitious systems [12,13,14,15], its application to CWM-based alkali-activated systems remains unexplored. Unlike conventional precursors (e.g., slag or fly ash) with relatively homogeneous chemical compositions, CWM is a heterogeneous material composed of crushed concrete, clay bricks, and mortar, resulting in complex multi-phase reactions during alkali activation. This study presents the first application of the KD model to CWM-based alkali-activated systems, with two key innovations: (1) parameter interpretation tailored to CWM’s unique mineralogy—the kinetic parameters (*K*_1_, *K*_2_, *K*_3_) are correlated with the specific phase composition of CWM (e.g., quartz, feldspar, and clay minerals) identified by XRD analysis; and (2) coupling of the KD model with thermogravimetric analysis (TGA)—the kinetic stages (NG, I, D) are validated against the thermal decomposition behavior of hydration products, providing a multi-technique framework for understanding the hydration mechanism of CWM-based binders.

To achieve these objectives, the experimental program was designed as follows. First, Portland cement (P.O.), Ca(OH)_2_ (CH), and water glass (WG) were used as activators for CWM, and the alkali activation scheme was optimized based on mechanical strength and activity index. Subsequently, hydration kinetics analysis was performed on the optimal mix proportion to establish the hydration kinetics equation of the CWM-based cementitious material. Finally, by integrating the hydration kinetics analysis with mechanical behavior characterization, the hydration and hardening mechanism of the alkali-activated CWM-based cementitious material was elucidated. The findings of this study are expected to provide a theoretical basis for material optimization and process improvement, enrich the fundamental understanding of building materials, and guide the development of novel CWM-based cementitious systems.

## 2. Materials and Methods

### 2.1. Raw Materials

#### 2.1.1. CWM

**Figure 1:**
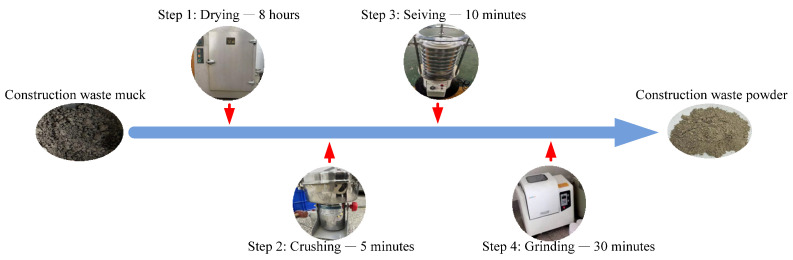
The preparation process of construction waste micro-powder (CWM).

**Figure 2:**
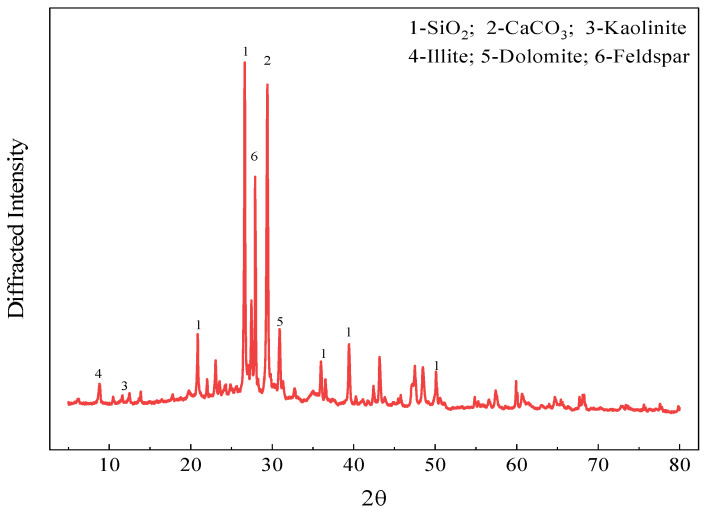
XRD pattern of CWM.

The CWM was sourced from Jiangsu Kangju Renewable Resources Technology Co., Ltd., Suqian, China. The raw waste muck was processed via crushing, screening, grinding, and drying, yielding a final product with a specific surface area of 1595 m^2^/kg. The processing flowchart is presented in Figure 1. This procedure followed the same method as described in previous works [16]. Table 1 summarizes the main chemical composition of the CWM. Table 2 reports the particle size distribution obtained from laser diffraction analysis. The main particles are those ranging from 1 to 20 micrometers in size, with a content of 78.38%. The CWM has a median particle size (D_50_) of 6.82 µm, with 10% of particles finer than 1.52 µm (D_10_) and 90% finer than 26.9 µm (D_90_). The uniformity coefficient (Cu = D_60_/D_10_) is approximately 6.3. The mineralogical composition, determined by XRD analysis (Figure 2), is presented in Table 3. Quartz (SiO_2_) is the most abundant phase, followed by feldspar and calcite (CaCO_3_). Kaolinite, illite, and dolomite are present as minor phases. The coexistence of these minerals reflects the complex origin of CWM, which contains both natural rock fragments and hydrated cement paste residues. The clay minerals (kaolinite and illite) are of particular interest, as they can be activated under alkaline conditions to form cementitious gels.

#### 2.1.2. Cement

The cement used in this study was P.O.42.5 grade ordinary Portland cement, produced by Suqian Sanxishui Cement Co., Ltd., Suqian, China. Its 28—day compressive strength was 45.2 MPa. The chemical composition is presented in Table 1.

#### 2.1.3. Alkali-Activator

Calcium hydroxide (CH): Produced by Xilong Co., Ltd., Shantou, China, of analytical purity, white powder, soluble in water and alkaline solutions. Silica gel (WG): Produced by Xilong Co., Ltd., Shantou, China, with a modulus (defined as the SiO_2_/Na_2_O molar ratio) of 2.3, AR (analytical purity), white powder.

#### 2.1.4. Experimental Water

Tap water from the municipal supply was used. All performance indicators met the relevant provisions of the “Concrete Mix Water Standard” (JGJ63-2006) [17].

### 2.2. Experimental Mixing Ratio

To evaluate the activation effects of different alkalis on the CWM recycled micro-powder, two series of mixtures were prepared. In the first series, nine mixtures were formulated by individually adding Portland cement (P.O.), calcium hydroxide (CH), or water glass (WG, sodium silicate) to the CWM micro-powder. In the second series, twelve mixtures were prepared by the simultaneous addition of two components: cement with CH, and cement with WG. The binding materials were CWM and P.O., with their masses being 500 g each and remaining constant. CH and WG were the alkaline activators, added as supplementary amounts to the total cementitious materials.

The mix proportions of the alkali-activated CWM system composite pastes are presented in Table 4. Specifically, the CWM replacement percentages (20%, 40%, and 50% by weight of the total binder) were selected based on preliminary optimization study: A series of trial mixes (10%, 20%, 30%, 40%, 50%) were tested to identify the range where compressive strength were not severely compromised. Percentages below 20% showed negligible mechanical improvement, while percentages above 50% led to a significant drop in strength. And the mixes were designed to achieve a target 28-day compressive strength of at least 20 MPa (for grouting applications). The CWM replacement levels were optimized within this constraint.

It should be noted that these are proportions for paste studies, with the water-to-binder ratio (*w*/*b*) as the key variable. The water content for each mix was determined as the standard consistency water requirement (according to GB/T 1346 [18]) to ensure a consistent and comparable initial fluidity across all samples, rather than targeting a specific workability for placement. This approach ensures that all pastes are evaluated starting from an equivalent and comparable rheological state, which is essential for a fair assessment of their subsequent hydration, setting, and strength development behaviors. The variation in total water content among mixes, therefore, serves as a direct indicator of the material’s inherent water demand, which is a key property influenced by the activation treatment of CWM.

### 2.3. Experimental Methods

#### 2.3.1. Laser Particle Size Analysis

The particle size distribution of the regenerated micro-powder was determined using a Malvern Mastersizer 3000 laser particle size analyzer (produced by Malvern Panalytical, Malvern, UK, measurement range: 0.02–3500 μm) in wet dispersion mode. The test conditions were as follows: deionized water was used as the dispersion medium (refractive index: 1.330); the particle refractive index was set to 1.710, and the absorption index (imaginary part) was 0.100. The Mie theory was applied as the scattering model, and the general analysis mode was selected. Prior to measurement, the samples were ultrasonically dispersed for 5 min, and the obscuration was controlled within the range of 5% to 15%. Each sample was measured three times, and the average value was reported. The particle size distribution was characterized by parameters including D_10_, D_50_, D_90_, span, and uniformity.

#### 2.3.2. X-Ray Diffraction Analysis

The mineral composition of CWM was characterized using X-ray diffraction (XRD) analysis pruduced by Shimadzu Corporation, Kyoto, Japan. XRD patterns were collected using a Rigaku D/max 2500PC X-ray diffractometer with Cu K_α_ radiation (λ = 0.15406 nm), operated at 40 kV and 30 mA. Data were recorded over a 2θ range of 5° to 90° with a step size of 0.02° and a scanning speed of 2°/min.

Prior to analysis, the CWM micro-powder samples were dried at 105 °C to constant weight and ground to a particle size of less than 75 μm (passing through a 200-mesh sieve). The powder was loaded into a glass sample holder, pressed and flattened with a glass slide to ensure a smooth surface flush with the holder rim. Care was taken to apply uniform pressure during sample preparation to minimize preferred orientation effects.

Phase identification was performed using Jade 6.0 software by comparing the obtained diffraction patterns with PDF standard cards. Semi-quantitative analysis of the mineral phases was conducted using the Rietveld full-spectrum fitting method to determine the content of each mineral phase.

#### 2.3.3. Adjustment of the Modulus of Sodium Silicate Solution

(1) Target modulus M1 = 1.0: Use a 10% concentration NaOH solution to directly dilute to the desired concentration. Slowly pour the prepared NaOH solution into the sodium silicate solution with a modulus of M_2_ = 2.3. The dripping time should be controlled at 18 min. After the dripping is completed, continue to stir for 30–60 min to ensure that Na_2_O is completely dispersed without local concentration differences. After the stirring is completed, determine the actual modulus by chemical titration and adjust it to the target modulus of 1.0.
(2) Target modulus M3 = 3.3: Use a 25% concentration silica sol colloid solution. Before use, mix it evenly to ensure there is no precipitation. Slowly add the silica sol to the sodium silicate solution, and the dripping time is 28 min. After the dripping is completed, continue to stir for 75 min to ensure that SiO_2_ is completely dispersed and forms a uniform sodium silicate solution. After the stirring is completed, determine the actual modulus by chemical titration and adjust it to the target modulus.

#### 2.3.4. Water Requirement Test of Normal Consistency for Alkali-Activated CWM-Cement

This is carried out in strict accordance with “Test Methods for Standard Water Content, Setting Time and Soundness of Cement” (GB/T 1346-2011) [18]. The specific steps are as follows:

(1) Preliminary water addition: Based on the fineness of the aggregate and cement, preliminarily estimate the water requirement at 400 mL. Accurately measure the water volume and pour it into the mixing bowl.
(2) Mixing of the paste: Weigh 500 g of mixtures and slowly add it to the water. Immediately start the mixer and stir at a speed of 140 ± 5 r/min for 120 s. Stop stirring for 15 s, and during this time, scrape the paste adhering to the inner wall and the blades into the center of the bowl. Restart the mixer and stir at a speed of 285 ± 10 r/min for 120 s. After mixing is complete, proceed immediately with the test.
(3) Testing with Vicat Apparatus: Place the mixed paste into the Vicat mold. Gently tap the mold 5–10 times with the mold rim, then use a straightedge to strike off the excess paste, making the surface flush with the mold rim. Place the mold on the base of the Vicat Apparatus. Adjust the test needle (diameter 1.13 ± 0.05 mm) so that it just contacts contact the surface of the paste. Suddenly release the test needle and allow it to freely sink into the paste. After 30 s, record the penetration depth.
(4) Judgment criteria: If the penetration depth of the test needle is 6 ± 1 mm, the corresponding water content is defined as the water requirement for standard consistency. If the depth is outside this range, adjust the amount of water, prepare a fresh paste, and repeat the procedure until the criterion is met.

#### 2.3.5. Sample Preparation, Curing and Strength Measurement

According to the standard ASTM C328 [19], the pure mortar samples were prepared. The specimen dimensions were 40 mm × 40 mm × 160 mm. After molding, the triple-gang mold was placed in a moist curing room and left to cure for 24 h. Then, the specimens were demolded and further cured in a moist cabinet at a temperature of (20 ± 1) °C and a humidity of ≥90%. After curing for 28 days, the specimens were tested for compressive strength, which was calculated using Formula (1).

(1) $$\sigma =\frac{F}{A}$$

where: σ is the compressive strength (MPa), F is the ultimate load (N), A is the compressive area (mm^2^).

#### 2.3.6. Activity Index Calculation of CWM

The compressive strengths of pure cement sample S0 and comparative alkali-activated CWM samples (S1–S21) were measured after 28 days of curing, and the activity index R was calculated based on Formula (2).

(2) $$R=\frac{R_{S}}{R_{C}}$$

*R_S_* represents the compressive strength of the test samples at the corresponding age, while *R_C_* represents the compressive strength of S0 ordinary Portland cement.

#### 2.3.7. Hydration Heat Test

The hydration heat release and heat release rate of alkali-activated CWM were determined using an isothermal conduction calorimeter (I-Cal 8000 HPC, Calmetrix, Boca Raton, FL, USA), which complies with ASTM C1702 [20]. A sample of 3–5 g was sealed in an ampoule with deionized water at a predetermined water-to-cement ratio. The ampoule was then placed in the calorimeter, and the internal mixer was activated. The heat flow was recorded continuously for up to 7 days, and the data were analyzed to determine the cumulative heat release and reaction kinetics.

#### 2.3.8. TGA Test

Thermogravimetric analysis (TGA) was performed on the samples using a DZ-TGA103 thermogravimetric analyzer (Nanjing Dazhan Testing Instrument Co., Ltd., Nanjing, China). The test was conducted under a nitrogen atmosphere at a flow rate of 50 mL/min, over a temperature range from 30 °C to 900 °C at a heating rate of 10 °C/min. After 3 and 7 days of curing, small fragments were taken from the paste samples, soaked in anhydrous ethanol to terminate hydration, and then ground into powder in a mortar with a pestle. The grinding process continued until the anhydrous ethanol had completely evaporated. The resulting powder was sieved through a 200-mesh sieve to ensure uniform particle size. The 3-day and 7-day hydration-terminated samples were then subjected to thermogravimetric analysis to determine their mass loss profiles and thermal decomposition behavior after the respective curing periods.

## 3. Results and Discussion

### 3.1. Activity Index

#### 3.1.1. Single Type of Alkali Activating CWM System

**Figure 3:**
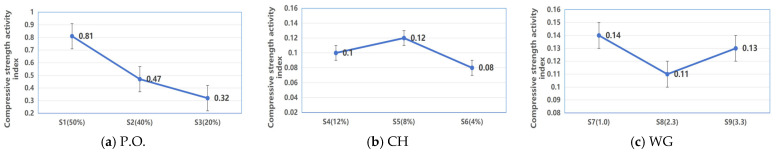
Compressive activity index of CWM under single-alkali activator: (**a**) P.O.; (**b**) CH; (**c**) WG.

Figure 3 presents the activity index of CWM activated by three single-type activators, highlighting how activator dosage or modulus affects reactivity and mechanical performance.

(1) P.O.

Figure 3a depicts the variation in the activity index of CWM when activated by Portland cement (P.O.) at dosages of 50% (S3), 60% (S2), and 80% (S1, mass fraction relative to total binder). A clear positive correlation between the P.O. dosage and the CWM activity index is observed: the index increases sequentially from 0.32 (S3) to 0.47 (S2), and reaches a maximum of 0.81 (S1).

This enhancement can be attributed to the dual roles of Portland cement:

① As a binder, P.O. provides a continuous matrix to encapsulate CWM particles, thereby reducing interfacial defects [21];

② As a chemical activator, the CH and silicate hydrates (C_3_S/C_2_S) hydrates released during P.O. hydration react with the amorphous SiO_2_ and Al_2_O_3_ in CWM (a typical pozzolanic reaction), generating additional C-S-H and C-A-S-H gels that densify the microstructure [22].

Correspondingly, the 28-day compressive strength of the S1 mixture reaches 45.6 MPa. This value not only meets the strength requirement for C40-grade concrete but also confirms the feasibility of utilizing high P.O. dosage CWM composites in structural applications, such as non-load-bearing walls or precast components.

(2) CH

For CH activation (Figure 3b), with dosages of 4% (S6), 8% (S5), and 12% (S4, mass fraction relative to CWM), the CWM activity index exhibits a “first increase then decrease” trend, which aligns with the optimal dosage effect of alkaline activators in pozzolanic systems [23].

The highest activity index (0.12) and corresponding 28-day compressive strength of 6.5 MPa are achieved at 8% CH (S5). However, when the dosage increases to 12% (S4), the activity index drops to 0.10, accompanied by a reduced strength of 5.8 MPa. This decline can be attributed to the excessive amount of free CH remaining in the matrix, which increases pore volume and weakens interfacial bonding [24]. Conversely, at the lower dosage of 4% (S6), the insufficient alkalinity (pH < 12) fails to effectively dissolve the silica–alumina phases in CWM, resulting in the lowest activity index (0.08) and compressive strength (4.3 MPa) among the three mixtures.

From an engineering perspective, the CWM-CH mixtures with CH dosages ranging from 4% to 8% yield compressive strengths comparable to those of graded subgrade soil. This makes them suitable for applications such as road subgrade filling. Considering both performance and cost, the 8% CH dosage is recommended as the optimal choice for achieving the best cost-effectiveness.

(3) WG

Figure 3c investigates the influence of the water glass (WG) modulus on the activity index of CWM. Tests were conducted at a constant WG dosage of 15% (by mass of CWM) with three moduli: 1.0 (S7), 2.3 (S8), and 3.3 (S9).

The results reveal a non-monotonic, “first decrease then increase” trend in the activity index with increasing modulus. The lowest index (0.11) was observed at the intermediate modulus of 2.3 (S8). In contrast, higher indices of 0.14 and 0.13 were achieved at the low modulus of 1.0 (S7) and the high modulus of 3.3 (S9), respectively.

This distinct trend can be attributed to the dual and competing effects of WG modulus on the system’s chemistry:

① At low modulus (M = 1.0), the high concentration of Na^+^ ions provides strong alkalinity, which effectively enhances the dissolution of silica–alumina phases from the CWM.

② At high modulus (M = 3.3), the system is rich in free SiO_2_. This abundant silica supply promotes the formation of additional C-S-H gels through reactions with calcium ions (Ca^2+^) released from CWM hydration or inherent impurities.

③ At the intermediate modulus (M = 2.3), the system strikes a suboptimal balance, providing neither sufficient alkalinity for effective dissolution nor an ample supply of reactive silica, leading to an insufficient pozzolanic reaction and thus the lowest activity index [25].

It is noteworthy that the maximum activity index achieved with WG activation (0.14 for S7) is substantially lower than that achieved with Portland cement (P.O.) activation (0.81 for S1, as shown in Figure 3a). This comparison clearly indicates that water glass alone is a less effective activator for CWM under the tested conditions, a finding that is consistent with prior research on alkali-activated construction waste materials [22].

#### 3.1.2. Double Alkali-Activated CWM System

(1). Composite Activation System of P.O. and CH

**Figure 4:**
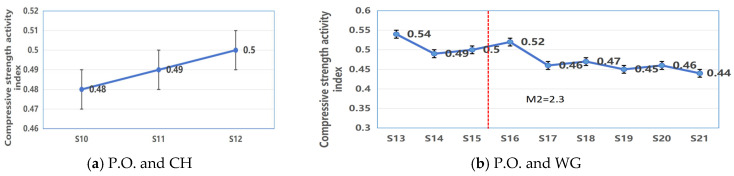
Compressive activity index of CWM under double-alkali activator: (**a**) P.O. and CH; (**b**) P.O. and WG.

As shown in Figure 4a, with the Portland cement (P.O.) dosage fixed at 60%, the compressive strength-based activity index of CWM exhibited a near-linear response to the addition of calcium hydroxide (CH). As the CH content increased from 4% (S10) to 12% (S12), the activity index increased by 0.02, corresponding to a slight compressive strength enhancement from 27.1 MPa to 28.0 MPa (a 3.3% increase). The maximum activity index was achieved at the 12% CH dosage, suggesting an optimal threshold for alkaline activation in this dual-alkali system. The proportional relationship between CH dosage and the modest strength gain implies a consistent but limited supplementary chemical activation mechanism provided by CH beyond the primary binding action of P.O.

The observed trend supports the hypothesis that CH enhances CWM reactivity primarily through two synergistic pathways within the P.O.-dominated system. First, it sustains a highly alkaline environment (elevated pH), which is known to accelerate the hydration of alite (the main component in P.O.) and, more critically for CWM, strongly promotes the dissolution of its amorphous silica–alumina phases, exposing more reactive sites. Second, the dissolved species subsequently engage in pozzolanic reactions with the calcium ions supplied abundantly by both the P.O. hydration and the added CH, leading to the formation of additional C-S-H and C-A-S-H gels that contribute to strength development [26].

(2). Composite Activation System of P.O. and WG

The compressive strength-based activity index of CWM exhibited a distinct nonlinear response to variations in both the dosage and modulus of the water glass (WG) activator, as summarized in Figure 4b. With the Portland cement (P.O.) content fixed at 60%, the following key trends were identified:

① Modulus-dependent attenuation: Increasing WG modulus from 1.0 to 3.3 resulted in a progressive decline in activity index (from 0.54 to 0.44) and compressive strength (from 30.2 MPa to 24.8 MPa). This inverse correlation aligns with prior studies, where higher modulus solutions accelerate silicate polymerization, reducing reactive hydroxyl availability [25].

② Optimal dosage windows: For all moduli (1.0, 2.3, 3.3), peak activity indices occurred at intermediate dosages within the range of 8–15%. The maximum indices achieved were 0.54, 0.52, and 0.46 for moduli 1.0, 2.3, and 3.3, respectively. Notably, the system with a modulus of 1.0 demonstrated superior activation efficiency, yielding a strength increase of +3.8 MPa compared to the P.O.-only reference system. This superior performance can be attributed to its more balanced alkalinity and slower gelation kinetics, which allow for a more controlled and complete reaction [27].

③ Synergistic enhancement: The dual-alkali systems, combining P.O. and WG, significantly outperformed systems modified with a single activator. A striking example is the combination of 1.0-modulus WG at an 8% dosage with P.O., which increased the compressive strength by 22.3 MPa. This represents a 273% enhancement relative to the strength achieved by WG alone under comparable conditions. This powerful synergy arises from complementary activation mechanisms: the Portland cement provides a sustained source of Ca^2+^ ions essential for the formation of C-S-H gel, while the water glass maintains a prolonged alkaline environment that promotes the continuous dissolution of amorphous silica–alumina phases from the CWM [28].

④ Competing mechanisms: The observed interplay between WG dosage and modulus suggests the influence of two competing factors. On one hand, beneficial effects dominate at low modulus (e.g., 1.0), where the activator optimally balances pH buffering and ion transport, thereby effectively enhancing the dissolution of slag-like components in the CWM. On the other hand, detrimental effects become prominent with high-modulus solutions (e.g., >3.0), which promote premature gelation and microstructure densification. This early gelation can physically encapsulate unreacted CWM particles and their reactive sites, limiting further reaction and ultimately compromising the final strength development.

#### 3.1.3. Discussion on the Activation of CWM by Alkali Activators

The activation efficiency of CWM was systematically evaluated using three alkali activators: P.O., CH, and WG. The experimental results not only quantified the performance differences among various systems, but also revealed the complex interplay mechanisms underlying them, providing a theoretical basis for the high value-added resource utilization of CWM.

(1) Performance of single-component alkali activation systems

In single-component activator systems, a clear performance gradient was observed. Portland cement (P.O.), serving as the activator, demonstrated unequivocal superiority. At an 80% dosage, it achieved an activity index of 0.81 and a corresponding compressive strength of 45.2 MPa. This outstanding performance is primarily attributed to the dual functional roles of P.O.: firstly, it acts as a binding matrix providing essential physical support and microstructure framework; secondly, its hydration process releases calcium hydroxide (CH) and various calcium silicate hydrates, which collectively supply the necessary calcium ions and maintain a persistently alkaline environment. This alkaline milieu is crucial for driving the pozzolanic reaction with the amorphous silica (SiO_2_) and alumina (Al_2_O_3_) phases present within the CWM.

In stark contrast, the efficacy of calcium hydroxide (CH) and water glass (WG) as standalone activators proved substantially limited. Their peak activity indices reached only 0.12 and 0.14, respectively. This empirical evidence strongly corroborates the foundational theory in alkali-activation chemistry: optimal performance requires the synergistic supply of both a high-alkalinity environment (to dissolve the solid precursors) and sufficient network-forming species (silicon, aluminum) to construct a stable gel matrix. Specifically, while CH alone can elevate the pH to effectively dissolve aluminosilicate phases from CWM, the resulting system suffers from a critical deficiency in soluble silicate, failing to form a robust, continuous gel network. Conversely, a low-modulus water glass (e.g., M = 1.0) can provide both alkalinity and reactive silicate, but the system is starved of the calcium ions essential for precipitating strength-giving calcium (alumino)silicate hydrate (C-(A)-S-H) gels in substantial quantities. Consequently, the structural strength development remains insufficient.

Therefore, from a practical application standpoint, the use of CH or WG as sole activators is more appropriate for scenarios where high mechanical strength is not a primary requirement, such as in certain road subbase filling applications. Their utility lies in scenarios demanding modest cementing properties coupled with the benefits of waste utilization.

(2) Analysis of the synergistic effect of composite alkali activation systems

The core finding of this study is the significant synergistic effect exhibited by the composite alkali-activation systems, which forms the focus of the discussion. Compared to systems with a single activator, both P.O.-CH and P.O.-WG composite systems achieved substantial performance enhancements. Notably, the P.O.-WG system (with 60% P.O. and 8% modulus-1.0 WG) showed a strength increase of up to 273% (+22.3 MPa) relative to the system activated by WG alone. This phenomenon cannot be attributed to a simple additive effect of the individual components but rather indicates a true synergistic interaction.

For the P.O.-CH system, the modest strength gain (0.3%) underscores the auxiliary role of CH within the P.O.-dominated matrix. The additional CH further elevates the pH of the pore solution, which may accelerate the hydration of alite (C_3_S) in Portland cement and provide a more sustained alkaline environment for the pozzolanic reaction. However, this benefit exhibits a threshold; excessive CH can lead to the aggregation of unreacted portlandite crystals at interfaces, potentially introducing structural defects.

In contrast, the P.O.-WG system demonstrates an exemplary chemical complementarity. The hydration of P.O. provides a steady and continuous supply of Ca^2+^ ions, which are crucial for forming the calcium silicate hydrate (C-S-H) gel skeleton. Concurrently, the introduction of low-modulus WG (n = 1.0) contributes Na^+^ to maintain high alkalinity—promoting the dissolution of the CWM—and supplies reactive silicate species. These silicate ions can rapidly react with the available Ca^2+^ to generate additional C-S-H gel and may also influence the morphology and distribution of early hydration products. This immediate coupling of a “calcium source” (from P.O.) and an “alkali-silicate source” (from WG) effectively overcomes the limitations inherent in single-activator systems. It achieves a synchronized optimization of the reaction kinetics and the quantity of binding phases, thereby explaining the dramatic leap in mechanical strength. This synergistic mechanism aligns with recent findings in alkali-activated slag or fly ash systems; however, this study is the first to clearly define the optimal ratio and quantify the enhancement magnitude for the P.O. and low-modulus WG combination within the complex, multiphase CWM system.

In summary, this research systematically elucidates the alkali-activation pathways for CWM, mapping the “activation-synergy” relationships across different activator combinations. This work enriches the theoretical foundation of alkali-activated cementitious material systems. The optimized composite formulation comprises cement and CWM as the binder, with 60 wt% ordinary Portland cement (P.O. 42.5) and 40 wt% CWM. Alkaline activators, namely a dosage of 8 wt% water glass, were added as external admixtures to the total binder mass. This formulation successfully balances activation efficiency, material cost, and solid waste utilization. It provides a directly applicable and referenceable formula for producing green concrete products with strength grades of C30 or higher, offering clear economic and environmental benefits.

### 3.2. Hydration Heat Characteristics and Comparative Stage Analysis of Alkali-Activated CWM System

**Figure 5:**
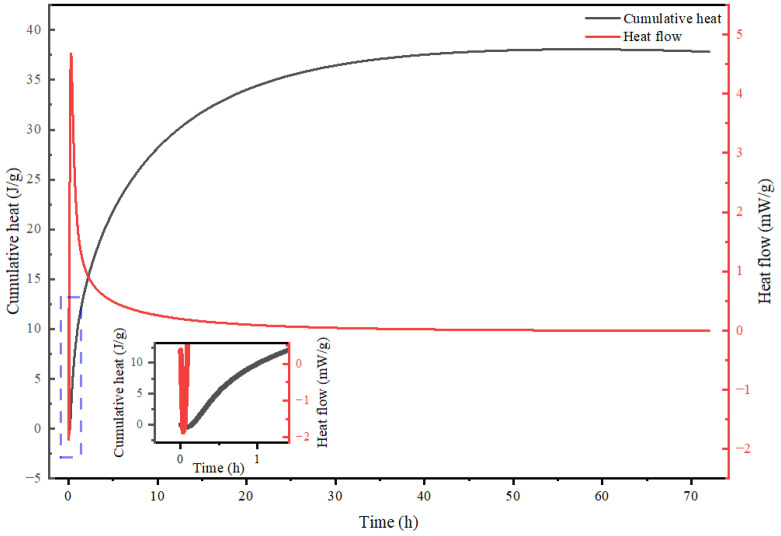
Cumulative heat curve and heat flow curve of CWM + WG system.

**Figure 6:**
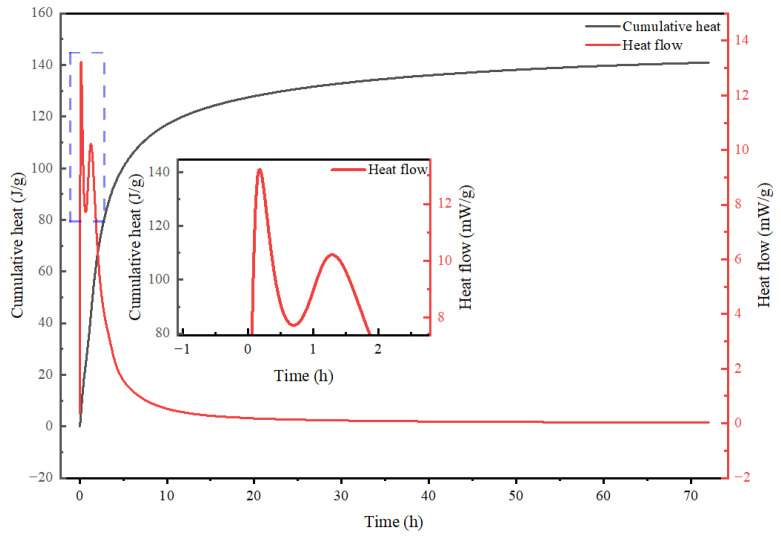
Cumulative heat curve and heat flow curve of CWM + WG + P.O. system.

The cumulative heat of hydration and the rate of heat release are critical parameters for investigating the hydration kinetics of hydraulic cementitious materials [29,30]. These thermodynamic variables serve as the foundation for describing system states and revealing reaction mechanisms, as well as predicting material properties such as strength development, shrinkage, and creep. For alkali-activated cementitious materials, the hydration process typically exhibits complex characteristics that differ significantly from ordinary Portland cement (OPC). As shown in Figure 5 and Figure 6, the hydration heat evolution profile of the alkali-activated CWM system is distinctly more multifaceted than the classic three-stage curve of OPC. Data acquired using a CALMETRIX I-CAL Flex high-performance isothermal calorimeter (I-Cal 8000 HPC, Calmetrix, Boca Raton, FL, USA) allow this process to be delineated into four typical stages, each corresponding to dominant reaction mechanisms and revealing fundamental contrasts with OPC hydration, as detailed below.

#### 3.2.1. Stage I: Initial Dissolution and Early Gel Nucleation—A Retarded Onset

Upon mixing, all alkali-activated CWM formulations exhibited a sharp exothermic peak (Figure 5 and Figure 6). However, a key distinction from OPC emerges immediately in the reaction onset kinetics. Compared to the rapid initial reaction of OPC (typically within ~15 min, dominated by C_3_A and C_3_S reactions) [31,32], the reaction onset in this system was significantly prolonged to approximately 9 min when using a water glass activator with a modulus of 1.0.

This retardation is attributed to a synergistic interplay of chemical and physical mechanisms induced by the alkaline activator and CWM. Low-modulus water glass (M_1_ = 1.0) provides a high concentration of OH^−^ ions, promoting rapid polymerization of silicate ions into a preliminary gel network. This nascent silicate-rich layer physically encapsulates cement and CWM particle surfaces, acting as a barrier that temporarily impedes water contact and retards the dissolution of reactive phases like C_3_S and C_3_A [33]. Concurrently, reactive aluminosilicate components in CWM compete with cement phases for essential ions (e.g., Ca^2+^, OH^−^), while inert particles act as physical fillers, diluting cement concentration and increasing diffusion path tortuosity [34,35]. The combined chemical inhibition and physical encapsulation lead to the pronounced prolongation of the initial reaction period, a phenomenon not observed in standard OPC hydration.

#### 3.2.2. Stage II: Superimposed Dissolution-Polycondensation—A Unique Thermal Signature

Following Stage I, the system enters a period absent in OPC hydration: a superimposed dissolution-polycondensation stage. Unlike OPC’s relatively simple induction period (1–4 h) [35], the alkali-activated CWM system displays a complex thermal signature where endothermic and exothermic processes compete. For the system activated with modulus-1.0 water glass, an endothermic peak is observed at approximately 0.18 h (Figure 6). This endothermic effect arises from energy-intensive processes: the continued dissolution and depolymerization of the aluminosilicate glass phase within CWM, requiring the breaking of strong Si-O and Al-O bonds, and potentially the recrystallization of early-formed gels [36]. This is superimposed on concurrent exothermic heat from the initial formation of primary gels and ongoing Portland cement hydration (C_3_S, C_2_S) [37,38]. The net heat flow is therefore subdued and variable, contrasting sharply with the singular, sharp exothermic acceleration of OPC’s silicate hydration. The characteristics of this stage are highly sensitive to activator chemistry; for instance, a higher modulus water glass introduces more polymerized silicate species, demanding more energy and time to depolymerize, thereby delaying and prolonging the endothermic peak [33].

This period represents a dynamic equilibrium between competing chemical pathways, fundamentally reflecting the synergistic interaction between traditional cement hydration and geopolymerization-like reactions. The Portland cement provides calcium ions and alkalinity, while the CWM and activator supply silicon and aluminum. Their simultaneous, competing reactions—each with distinct thermodynamic signatures—give rise to this unique calorimetric profile, underscoring the sophisticated kinetics of multi-component alkali-activated systems.

#### 3.2.3. Stage III: Main Polycondensation and Sustained Acceleration—A Prolonged Main Reaction

Subsequently, the system transitions into the main reaction period, characterized by a pronounced and sustained exothermic peak (Figure 5 and Figure 6). This stage marks the accelerated precipitation and polycondensation of primary binding gels, predominantly C-(A)-S-H type, which constitute the core strength-developing reaction. Here, another stark contrast with OPC is evident in the reaction duration. While OPC’s main reaction (alite/C_3_S hydration) typically concludes within 4 to 8 h [39], the acceleration period in the alkali-activated CWM system is markedly extended to approximately 20 h (for M = 1.0). This significant prolongation is driven by sustained alkaline activation, which continuously stimulates the dissolution of CWM components and gel polycondensation, preventing the rapid reactant depletion that limits kinetics in OPC systems.

The underlying mechanism involves a continuous, synergistic supply of reactive species. The alkaline activator maintains depolymerization of CWM glass, releasing silicate and aluminate monomers, while ongoing Portland cement hydration provides a steady stream of calcium ions [40,41]. The combination of these three components facilitates continuous nucleation and growth of C-(A)-S-H gel, responsible for the sustained exothermic heat release. This extended period of high activity is a direct consequence of the multiphase alkali-activation process, differing fundamentally from the more self-limiting OPC hydration.

#### 3.2.4. Stage IV: Deceleration Period—Diffusion Control Governed by Microstructure

Finally, following the peak heat release rate, the reaction enters a deceleration period (evident after ~50 h in Figure 6). This stage marks a transition from chemical reaction control to diffusion control, a mechanistic step also present in OPC but occurring on a different timeline due to the preceding prolonged reactions.

The shift is caused by progressive microstructural densification. As C-(A)-S-H gel continuously precipitates and deposits on unreacted particles, it forms an increasingly thick, dense, and interconnected encapsulation layer [26,42]. This growing physical barrier significantly impedes the transport of essential ions (Ca^2+^, OH^−^, silicate/aluminate species) to the reaction fronts. Consequently, the supply of fresh reactants becomes rate-limiting. The heat release rate thus steadily declines as the system approaches a quasi-stable state governed by slow solid-state diffusion processes, completing the hydration sequence.

#### 3.2.5. Summary of Distinctive Hydration Features

In summary, the hydration of the alkali-activated CWM system is characterized by a more complex and protracted sequence compared to OPC, as revealed by isothermal calorimetry. The key distinguishing features, stemming from the multiphase reactions between the alkaline activator and heterogeneous CWM, are:

(1) A Retarded Onset (Stage I), due to chemical and physical inhibition mechanisms not found in OPC.
(2) A Unique Superimposition Stage (Stage II), where competing endothermic and exothermic processes create a complex thermal signature absent in OPC’s simple induction period.
(3) A Prolonged Main Acceleration Period (Stage III), driven by sustained alkaline activation, contrasting with OPC’s shorter reaction window limited by reactant depletion.
(4) A Diffusion-Controlled Deceleration (Stage IV), governed by microstructural densification, which shares a common mechanistic endpoint with OPC but is reached via a distinct kinetic pathway.

### 3.3. Thermogravimetric Analysis of Alkali Activated CWM

Thermogravimetric analysis (TGA) is a crucial and quantitative method for investigating the composition and thermal stability of hydration products in cement-based materials. By measuring the mass change of a sample under a programmed temperature profile, TGA provides insights into physical phenomena (e.g., evaporation, sublimation) and chemical phenomena (e.g., dehydration, decomposition, oxidation). For alkali-activated CWM systems, their thermal decomposition behavior exhibits distinct stage-wise characteristics and pronounced age-dependent trends, as illustrated in Figure 6. This complexity arises from the multiphase reactions between the alkaline activator and the heterogeneous CWM components, which differ fundamentally from the hydration of ordinary Portland cement.

#### 3.3.1. Thermal Decomposition Behavior of the 3-Day Cured Sample

**Figure 7:**
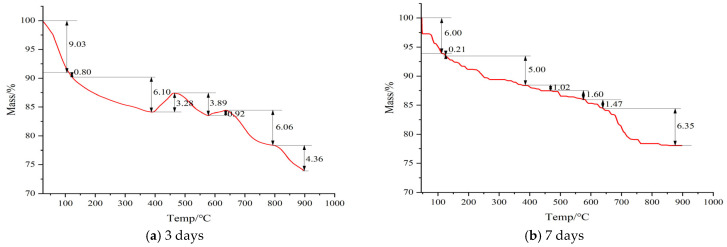
TG curve of CWM activated by a dual-alkali solution at curing ages of (**a**) 3 days and (**b**) 7 days.

The TG curves for the 3-day cured sample (Figure 7a) reveal a multi-stage decomposition process, reflecting the complex and evolving nature of the hydration products in this early age.

(1) Low-Temperature Mass Loss (<388 °C)

The mass loss was 15.87%, mainly attributed to: 9.03% evaporation of free water (<110 °C), 0.80% decomposition of aluminosilicate phases (e.g., AFt, AFm) (<120 °C) [43], and 6.10% dehydration of C-S-H gel (120–380 °C) [44].

(2) Anomalous Mass Increase (388~465 °C)

A 3.28% mass increase was observed, which is uncommon in ordinary Portland cement. This phenomenon may indicate secondary hydration or phase transformation reactions, such as the reaction of residual active Si/Al components with Ca(OH)_2_ at high temperatures to form additional C-S-H gel, or the recrystallization of calcium aluminosilicate minerals [45].

(3) Calcium Hydroxide Decomposition (465~574 °C)

The mass loss was 3.89%, corresponding to the decomposition of Ca(OH)_2_ into CaO and H_2_O. The actual mass loss rate was much lower than the theoretical value of pure Ca(OH)_2_ (24.3%) [43], suggesting that most of the Ca(OH)_2_ had undergone pozzolanic reactions with CWM within the 3-day curing period, generating C-S-H gel [46].

(4) Minor Mass increase in the high-temperature range (574~637 °C)

A 0.92% mass increase may be related to high-temperature solid-phase reactions involving iron phases or the decomposition/formation of metastable carbonate intermediate products [47].

(5) High-temperature decomposition (>637 °C)

The mass loss in this stage was 10.40%, which mainly attributed to the decomposition of carbonate minerals (such as CaCO_3_) [47]. The actual mass loss rate was lower than the theoretical value of pure CaCO_3_ (44.0%), suggesting that the carbonate content in CWM was limited or that the decomposition was incomplete.

#### 3.3.2. Thermal Decomposition Behavior of the 7-Day Cured Sample

The TG curve of the 7-day cured specimen (Figure 7b) shows a continuous mass loss without anomalous weight gain, suggesting a more advanced hydration state compared to earlier ages [48].

The mass loss profile can be divided into three main temperature intervals:

- <388 °C: Mass loss of 11.21%, mainly attributed to dehydration of C-S-H gel and aluminate-containing phases (e.g., AFt, AFm) [45].
- 388~574 °C: Mass loss of 2.62%, corresponding to decomposition of residual portlandite (Ca(OH)_2_). The low mass loss suggests effective consumption of Ca(OH)_2_ through pozzolanic reactions [44].
- >574 °C: Mass loss of 7.82%, attributed to combined decomposition of C-(A)-S-H gels and carbonate species (e.g., CaCO_3_) [39].

#### 3.3.3. Thermal Behavior Contrastive Analysis of the 3-Day and 7-Day Cured Sample

Based on Table 5, Total mass loss decreased from −26.02% (3 days) to −21.65% (7 days), a reduction of 4.37%, indicating a denser microstructure and increased bound water content with prolonged curing.

- Free Water (<110 °C): Mass loss decreased from −9.03% to −6.00% (+3.03%), confirming conversion of free water to chemically bound water.
- AFt (100–120 °C): Decreased from −0.80% to −0.21% (+0.59%), suggesting transformation of ettringite to more stable phases.
- C-S-H Gel (120–388 °C): Slight decrease from −6.10% to −5.00% (+1.10%), reflecting gel densification.
- Anomalous Region (388–465 °C): A mass gain of +3.28% at 3 days shifted to −1.02% mass loss at 7 days (−4.30%), indicating consumption of reactive Si/Al species and disappearance of high-temperature solid-state reactions.
- Ca(OH)_2_ (465–574 °C): Decreased from −3.89% to −1.60% (+2.29%), confirming ongoing pozzolanic consumption of portlandite.
- High-Temperature Region (574–637 °C): Mass gain of +0.92% at 3 days shifted to −1.47% mass loss at 7 days (−2.39%), consistent with the depletion of reactive components.
- CaCO_3_ (>637 °C): Decreased from −10.40% to −6.35% (+4.05%), possibly due to encapsulation by hydration products or carbonate consumption in the alkaline environment.

The above-mentioned results demonstrated the evolution of thermal decomposition with curing age and confirmed the effectiveness of the composite activation mechanism for CWM:

(1) Alkaline activation: sodium silicate provides a sustained alkaline environment, which promotes the continuous dissolution of silicon and aluminum species from the CWM.
(2) Ion exchange and polycondensation: the exchange among Na^+^, Ca^2+^, and other cations facilitates the formation of a dense network of cementitious gels (C-S-H, C-A-S-H, N-A-S-H), leading to the observed improvement in microstructural homogeneity and thermal stability.

## 4. Hydration Kinetics Characteristics of Alkali-Activated CWM

### 4.1. Hydrodynamic Model

#### 4.1.1. Model Selection

The hydration process of alkali-activated CWM exhibits distinct multi-stage characteristics, indicative of shifting dominant mechanisms over time. To quantitatively describe such kinetics, several mathematical models commonly employed for cementitious materials are evaluated. These include the Johnson-Mehl-Avrami-Kolmogorov (JMAK) model [49,50,51,52,53], which primarily describes nucleation and growth processes; the phase boundary reaction model [54]; the diffusion-controlled growth model [55]; and the comprehensive Krstulović–Dabić (KD) model [56]. The KD model is particularly relevant as it conceptualizes the overall hydration process as the combined result of three consecutive stages: nucleation and crystal growth (NG), phase boundary reaction (I), and diffusion (D), each governed by a distinct mechanism. A comparative summary of the key features, governing equations, and applicable stages of these models is provided in Table 6.

The Krstulović–Dabić (KD) model is distinguished by its foundation in physical and chemical mechanisms, rather than relying solely on empirical fitting. This mechanistic basis ensures that the model parameters carry clear physical significance. For instance, the rate constants *K*_NG_, *K*_I_ and *K*_D_ represent the reaction rates of the nucleation and growth (NG), phase boundary reaction (I), and diffusion (D) stages, respectively. Meanwhile, the exponent n is related to the geometric characteristics of the nucleation and growth of hydration products. Due to this mechanistic framework, the KD model is particularly well-suited for describing the complex, multi-stage hydration kinetics of cementitious materials [57].

The model conceptualizes the overall hydration process as three consecutive, potentially overlapping stages, each controlled by a distinct mechanism. The corresponding kinetic equations are presented in Equation (3). A fundamental principle of this model is that the overall hydration rate at any time is determined by the minimum value among the rates of these three individual stages. In other words, the kinetics are governed by rate-limiting step (the slowest among the three) at any given point during the reaction.

(3) $$\frac{d\xi }{dt}=\min(\frac{d\xi }{{dt}_{NG}},\frac{d\xi }{{dt}_{I}},\frac{d\xi }{{dt}_{D}})$$

The three consecutive stages of the KD model are specifically described as follows:

Stage 1: Nucleation and Growth (NG). This initial stage is dominated by the energy barrier for nucleation of new phases. The kinetics follow the Avrami equation, which describes phase transformation under nucleation and growth control. The corresponding rate and degree of hydration equations are given by Equations (4) and (5), respectively.

(4) $${\left[-In\left(1-\alpha \right)\right]}^{\frac{1}{n}}=K_{NG}(t-t_{0})$$

(5) $$\frac{d\alpha }{dt}=K^{\prime }n\left(1-\alpha \right){\left[-In\left(1-\alpha \right)\right]}^{\frac{n-1}{n}}$$

Stage 2: Phase Boundary Reaction (I). In this stage, the process is controlled by the chemical reaction rate at the interface between the unreacted core and the hydration product layer. Under the assumption of a constant reaction area, it exhibits linear kinetics. The governing equations for this stage are presented as Equations (6) and (7).

(6) $${\left[1-\left(1-\alpha \right)\right]}^{\frac{1}{3}}=K_{I}(t-t_{0})$$

(7) $$\frac{d\alpha }{dt}={K^{\prime }}_{I}•3{\left(1-\alpha \right)}^{\frac{2}{3}}$$

Stage 3: Diffusion (D). The final stage is determined by the diffusion rate of reactants (e.g., water, ions) through the increasingly thick product layer to the reaction front. This diffusion-controlled process is typically described by equations derived from solutions to Fick’s second law for specific particle geometries, such as spherical grains. The kinetic expressions for this stage are shown in Equations (8) and (9).

(8) $${\left[1-\left(1-\alpha \right)\right]}^{\frac{2}{3}}=K_{D}(t-t_{0})$$

(9) $$\frac{d\alpha }{dt}={K^{\prime }}_{D}•3{\left(1-\alpha \right)}^{\frac{2}{3}}[{2-2\left(1-\alpha \right)}^{\frac{1}{3}}]$$

Here: *K*_NG_′, *K*_I_′, *K*_D_′ are the apparent reaction rate constants for the nucleation and growth (NG), phase boundary reaction (I), and diffusion (D) processes, respectively. *t*_0_ denotes the time at the end of the induction period, marking the onset of the main acceleration period. *α* are critical degrees of hydration that signify the transitions between consecutive stages. Specifically, *α*_1_ represents the hydration degree at which the process shifts from being dominated by nucleation and growth (NG) to being controlled by the phase boundary reaction (I). Similarly, *α*_2_ corresponds to the transition from the phase boundary reaction (I) to the diffusion-controlled (D) stage.

#### 4.1.2. Determination of Model Parameters

The determination of the kinetic parameters for the Krstulović–Dabić (KD) model relies on several foundational quantities: the theoretical maximum heat of hydration (*Q*_max_), the hydration degree (α), and the hydration rate (dα/dt). These are calculated sequentially using Equations (10), (11) and (12), respectively.

(10) $$\alpha =\frac{Q(t)}{Q_{max}}$$

(11) $$\frac{d\alpha }{dt}=\frac{dQ}{dt}∙\frac{1}{Q_{max}}$$

(12) $$\frac{1}{Q(t)}=\frac{1}{Q_{max}}+\frac{t_{50}}{Q_{max}(t-t_{0})}$$

The variables and parameters involved in these calculations are defined as follows:

*t* is the hydration time. *t*_0_ is a critical time parameter defined as the time at the end of the induction period, which marks the onset of the main acceleration stage. In practice, *t*_0_ is often determined from the intersection point of the differential curve of the cumulative heat. *Q*(*t*) is the cumulative heat released at time *t*. *Q*_max_ is the theoretical maximum cumulative heat release upon complete hydration. It is typically determined by extrapolating experimental data. *t*_50_ represents the hydration time required for the heat release to reach 50% of *Q*_max_, analogous to a half-life in reaction kinetics. α denotes the degree of hydration, which is fundamentally linked to the heat release by the relationship α = *Q*(*t*)/*Q*_max_.

The kinetic parameters of the Krstulović–Dabić (KD) model were performed using nonlinear least-squares regression in OriginPro 2024b, and the goodness of fit was evaluated using the coefficient of determination (R^2^).

First, the experimentally measured time-dependent data of cumulative hydration heat (*Q*(*t*)) and heat release rate (*dQ*/*d*t) were fitted using Equation (10). This fitting process, involving a linear regression of a transformed plot (1/*Q* vs. 1/(*t* − *t*_0_)), yields the theoretical maximum hydration heat (*Q*_max_) by extrapolation to infinite time.

Subsequently, the obtained *Q*_max_ value was substituted into Equations (11) and (12) to calculate the hydration degree (α) and the instantaneous hydration rate (dα/dt) for each time point, effectively normalizing the calorimetric data into kinetic variables.

Finally, these derived α and dα/dt values were used as inputs to the core kinetic equations of the KD model (Equations (4)–(9)). A non-linear least-squares fitting routine was employed to optimize the model parameters. Given the complexity of the model and the potential for local minima, a robust optimization algorithm such as the Levenberg-Marquardt algorithm was utilized. In some methodologies, global optimization techniques like particle swarm optimization (PSO) are recommended to ensure convergence to a biologically or physically plausible global optimum and to assess parameter identifiability. This final fitting step directly yielded the key hydration kinetic parameters: the exponent n (related to nucleation geometry), and the rate constants for the three consecutive stages, *K*_NG_, *K*_I_, and *K*_D_.

**Figure 8:**
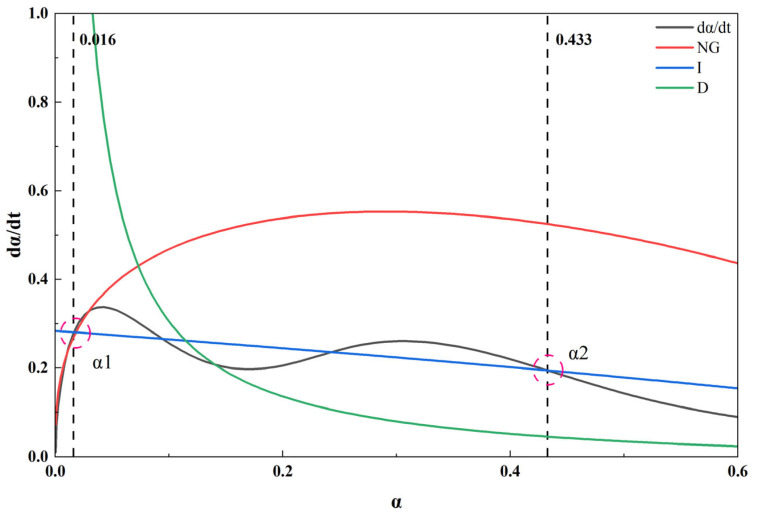
Stage-wise decomposition of the hydration kinetics by the KD model fitting.

The kinetic parameters derived from the Krstulović–Dabić (KD) model and listed in Table 7 were utilized to plot the hydration rate (dα/dt) against the hydration degree (α), as shown in Figure 8. The resultant curves clearly demonstrate that the hydration kinetics of the alkali-activated CWM system conforms to the three-stage KD model. The fitting results for each stage are as follows:

- NG stage: t_0_ = 0.01 h; n = 1.52144; *K*_NG_ = 0.0244. NG uses the acceleration period (0.05–0.15 h), with a R-squared value of 0.99955.
- I stage usually occurs during the decline period after the peak, and I uses Time = 0.21667 to 0.5; *K*_I_ = 0.09464, with a R-squared value of 0.99709.
- D stage: *K*_D_ = 0.00753, using Time = 20 to 50, with a R-squared value of 0.99987.

### 4.2. Analysis of Influencing Factors on Hydration Dynamics of Alkali-Activated CWM

The kinetic parameters derived from the Krstulović–Dabić (KD) model and listed in Table 7 were utilized to plot the hydration rate (dα/dt) against the hydration degree (α), as shown in Figure 8. The resultant curves clearly demonstrate that the hydration kinetics of the alkali-activated CWM system conforms to the three-stage KD model. This conformity indicates that the hydration process is not governed by a single mechanism throughout but undergoes sequential transitions in the rate-controlling steps as the reaction proceeds.

The obtained stage-specific kinetic parameters (*K*_NG_, *K*_I_, *K*_D_) reveals the following mechanistic evolution:

(1) Initial Acceleration Period: Dominated by Nucleation and Crystal Growth (NG).

In this initial stage, the high alkalinity provided by the activator rapidly depolymerizes the vitreous structure of the CWM, releasing active cations (e.g., Ca^2+^, Al^3+^) and silicate anions into the solution. Once the ionic concentration exceeds the supersaturation threshold for hydration products (primarily C-(A)-S-H gel), heterogeneous nucleation occurs, followed by rapid crystal growth. This leads to a sharp increase in the reaction rate, quantified by the rate constant *K*_NG_ [51,52]. The Avrami exponent n, associated with this stage, reflects the geometry of the nucleation and growth process.

(2) Mid-term Deceleration Period: Controlled by Phase Boundary Reaction (I).

As hydration products continuously precipitate and form a coherent layer coating the unreacted particle surfaces, the reaction enters the I stage. The rate at this point is governed by the chemical reaction kinetics at the interface between the product layer and the fresh CWM core. The physical presence of this layer begins to hinder the direct contact of reactants, causing the overall reaction rate to decelerate. The rate constant *K*_I_ characterizes the intrinsic speed of this interfacial chemical reaction. The properties of this interfacial layer, influenced by the activator type (e.g., through its effect on the density of the interfacial transition zone), play a crucial role in determining *K*_I_ [58].

(3) Later Stable Period: Governed by Diffusion (D).

When the hydration product layer grows sufficiently thick and dense, the transport of reactant ions (e.g., Ca^2+^, OH^−^) through this layer becomes the slowest step. The reaction thus transitions to diffusion control. The ion diffusion rate through the increasingly impermeable C-S-H gel network is significantly lower than the preceding chemical reaction rates, leading to a slow, stable hydration period characteristic of long-term strength development [42,43]. The rate constant *K*_D_ in this stage is a direct measure of the diffusional resistance, which is influenced by the microstructure of the product layer, such as its porosity, pore size distribution, and the Ca/Si ratio of the C-(A)-S-H gel.

The observed sequential transition from NG to I and finally to D control is a direct kinetic signature of the microstructural evolution within the cementitious matrix. The continuous formation and accumulation of hydration products progressively reduce the overall porosity and increase the tortuosity of the pore network. This microstructural densification elevates the resistance to ion migration, thereby shifting the dominant mechanism from fast, chemically controlled processes (NG, I) to the slow, physically limited diffusion process (D). The critical hydration degrees *α*1 and *α*2, marking these transitions, are thus key indicators linking the macroscopic hydration kinetics to the underlying microstructural development.

## 5. Discussion

### 5.1. Understanding the Hydration Kinetic Parameters of Alkali-Activated CWM

A profound understanding of the physical significance behind the kinetic parameters of the Krstulović–Dabić (KD) model is crucial for interpreting the hydration behavior of alkali-activated CWM. Each parameter provides a quantitative lens into a distinct mechanistic regime of the complex reaction process.

**Figure 9:**
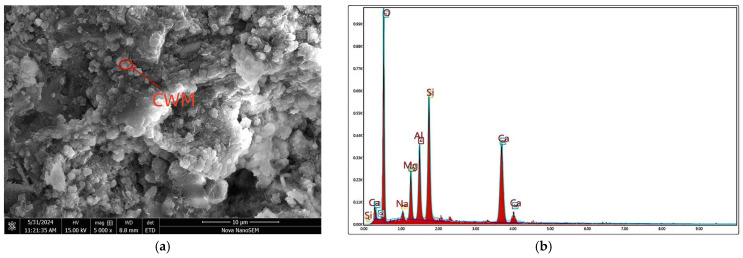
(**a**) CWM particles as additional nucleation sites. (**b**) Spectrogram of CWP particles.

The rate constant of the nucleation and growth stage (*K*_NG_) primarily reflects the initial rate of formation and growth of early hydration products. This rate is highly sensitive to the ionic supersaturation in the pore solution, which is governed by the dissolution kinetics of the CWM under alkaline attack. A higher *K*_NG_ indicates a more rapid release of reactive Si and Al species from the CWM, leading to faster precipitation of C-(A)-S-H or N-A-S-H gels. In composite systems, fine CWM particles can act as additional nucleation sites, potentially accelerating early hydration and enhancing the *K*_NG_ value, as illustrated conceptually in Figure 9.

**Figure 10:**
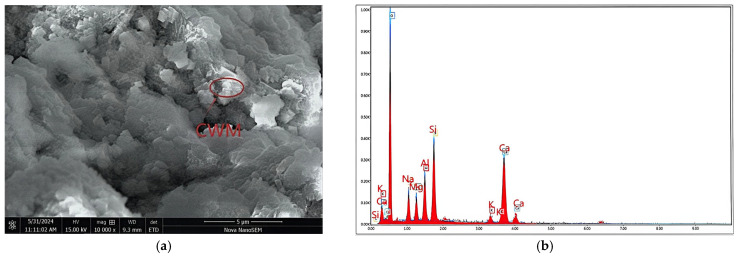
(**a**) Dense or passivating layer on the CWM particles surface. (**b**) Spectrogram of CWP particles surface.

The rate constant of the phase boundary reaction stage (*K*_I_) represents the intrinsic chemical reaction rate at the interface between the unreacted CWM core and the surrounding hydration product layer. This parameter is influenced by the specific surface area and the surface reactivity of the CWM particles. However, it is also modulated by the nature of the initial product layer formed. The formation of a dense or passivating layer on the particle surface, which can be influenced by the activator type or the presence of certain admixtures, may reduce the effective *K*_I_ by hindering the interfacial reaction, as illustrated in Figure 10.

The rate constant of the diffusion stage (*K*_D_) characterizes the rate at which reactant ions (e.g., OH^−^, Na^+^, silicate species) diffuse through the increasingly thick and dense hydration product layer to sustain the reaction. This parameter is fundamentally linked to the microstructural density and tortuosity of the gel matrix. An intriguing hypothesis in CWM-containing systems is that the resulting C-(A)-S-H or hybrid gels may possess a more open or less dense nanostructure compared to those in conventional alkali-activated slag systems. This structural difference could lower the resistance to ion diffusion, manifesting as a higher apparent *K*_D_ value. The ultimate microstructure, and hence *K*_D_, is a result of the complex interplay between dissolution, gel precipitation, and polycondensation processes, which can be altered by the chemical and mineralogical composition of the CWM [57,58].

The stage transition points *α*1 and *α*2 serve as two pivotal characteristic parameters in the Krstulović–Dabić (KD) model, quantitatively demarcating the shifts in the rate-controlling mechanisms during hydration. Physically, *α*1 represents the degree of hydration at which the initially formed gel nuclei have grown to cover the particle surfaces, causing the rate-controlling mechanism to shift from nucleation and growth (NG) to phase boundary reaction (I). In other words, *α*1 marks the point at which the reaction front transitions from being controlled by the formation of new nuclei to being controlled by the inward movement of the reaction interface. Conversely, *α*2 represents the degree of hydration at which the hydration product layer becomes sufficiently thick that ion transport through this layer becomes the rate-limiting step, marking the transition from phase boundary reaction (I) to diffusion control (D). At this point, the reaction kinetics shift from chemical reaction control to mass transport control.

In alkali-activated CWM systems, the *α*1 value is typically found to be 0.016, as illustrated in Table 8. This extremely low *α*1 value indicates that the NG stage is very short, suggesting that the early formation of hydration product nuclei is highly efficient and rapidly saturates the available particle surfaces. Compared to other cementitious systems listed in Table 8, the *α*1 value of 0.016 for alkali-activated CWM is significantly lower than those reported for ordinary Portland cement (0.145–0.267) [59,60], slag-cement blends (0.070–0.195) [59,61], Brick-Cement [62] and alkali-activated slag (0.149–0.253) [27,63]. This comparison quantitatively demonstrates that the nucleation barrier in the CWM system is substantially lower, and the transition to interface-controlled reaction occurs at a much earlier stage of hydration. This efficiency can be attributed to the alkaline activator rapidly depolymerizing the reactive phases within CWM, leading to a swift rise in ionic supersaturation and subsequent precipitation.

In contrast, the *α*2 value 0.433 exhibits significant sensitivity to the CWM content and, more importantly, the alkali-activation conditions. This value indicates that the phase boundary reaction (I stage) controls the hydration kinetics up to approximately 43% hydration, after which diffusion becomes rate-limiting. Notably, as shown in Table 8, the *α*2 value of 0.433 for alkali-activated CWM is substantially higher than those reported for ordinary Portland cement (0.232–0.332), slag-cement blends (0.180–0.311), and alkali-activated slag (0.218–0.346). This comparison reveals a unique characteristic of the CWM system: the phase boundary reaction stage is prolonged, and the transition to diffusion control is delayed to a higher degree of hydration. This may be attributed to the continuous supply of reactive Si and Al species from CWM, which sustains the phase boundary reaction and delays the formation of a dense diffusion barrier.

For instance, research on weakly alkaline-activated slag cements has shown that compositions favoring higher *α*1 and *α*2 values correspond to a prolonged duration of both NG and I stages [48]. This phenomenon is mechanistically linked to the rate of glass phase depolymerization, which is modulated by the alkalinity and the type of activator used. The choice of activator (e.g., sodium hydroxide, sodium silicate) not only influences the early dissolution but also dictates the nature and density of the resulting hydration products, thereby affecting when the diffusion barrier becomes rate-limiting (i.e., the value of *α*2) [58,59]. Furthermore, the incorporation of CWM, which initially behaves as an inert filler but participates in pozzolanic reactions at later ages, can alter the microstructure development and thus impact the transition points. A comprehensive comparison of these kinetic parameters across different systems is essential to elucidate the unique role of CWM.

### 5.2. The Design of Alkaline Activation CWM

Owing to its inherent capacity for multi-stage mechanism identification and the clear physical significance of its parameters, the Krstulović–Dabić (KD) model has emerged as a powerful and widely adopted tool for investigating the hydration kinetics of alkali-activated materials, including those incorporating CWM. The model conceptually decomposes the complex hydration process into three fundamental, consecutive stages: nucleation and crystal growth (NG), phase boundary reaction (I), and diffusion (D). This tripartite framework aligns remarkably well with the actual hydration characteristics observed in alkali-activated CWM systems, where an initial rapid reaction is followed by deceleration and a final stable period.

The true strength of the KD model lies in its quantitative nature. By analyzing the derived kinetic parameters—the rate constants for each stage (*K*_NG_, *K*_I_, *K*_D_), the Avrami exponent (n) related to nucleation geometry, and the critical transition points between stages (*α*1, *α*2)—it becomes possible to decipher the influence mechanisms of various factors on the hydration process. For instance, studies on systems with supplementary cementitious materials have shown how factors like the properties of the additive can alter these parameters: limestone, Fly Ash was found to increase the rate constants [64]. Similarly, in alkali-activated CWM systems, parameters such as the sodium silicate content and modulus, cement content, and CWM content itself can be systematically evaluated through their impact on these kinetic descriptors, providing a theoretical roadmap for material optimization.

The hydration heat characteristics—a direct output from isothermal calorimetry which serves as the primary data source for KD model analysis—of the alkali-activated CWM system are profoundly influenced by the synergy between the ordinary Portland cement (OPC) content and the modulus of the sodium silicate activator. For a given OPC content (e.g., 60%), a low-modulus sodium silicate (e.g., modulus 1.0) provides a higher effective alkalinity. This aggressively depolymerizes the CWM, potentially accelerating the early dissolution and nucleation (affecting *K*_NG_). However, it may also promote the formation of a denser initial product layer, thereby strengthening the physical diffusion barrier at later ages (potentially lowering *K*_D_). Conversely, a high-modulus sodium silicate (e.g., modulus 2.3) supplies more polymeric silicate species. These can directly integrate into the forming gel network, leading to the development of a more stable but possibly slower-forming silicate structure, which could manifest as an extended reaction duration across the later stages. This complex, modulus-dependent kinetic behavior underscores the critical role of activator chemistry and provides a vital theoretical foundation for the design of performance-tunable, alkali-activated cementitious materials.

### 5.3. Novelty and Contribution

Application to CWM: The first KD model analysis of CWM-based alkali-activated systems, addressing the unique challenges posed by CWM’s heterogeneous mineralogy.

Coupling with TGA: A novel multi-technique framework that validates the kinetic stages (NG, I, D) against thermal decomposition behavior, providing complementary evidence for the hydration mechanism.

Parameter interpretation: A CWM-specific interpretation framework that correlates kinetic parameters (*K*_1_, *K*_2_, *K*_3_) with the dissolution, phase boundary reaction, and diffusion processes unique to CWM systems.

## 6. Conclusions and Outlook

### 6.1. Main Conclusions

Based on a comprehensive investigation into the alkali activation of CWM, the main conclusions of this study are summarized as follows:

(1) Optimized Activator Formulation for Enhanced Reactivity. The activating efficacy on CWM follows the order: composite activator > silicate cement > Ca(OH)_2_ ≈ sodium silicate alone. A synergistic effect was achieved with a composite activator, leading to the identification of an optimal formulation: 40% CWM, 60% silicate cement, and 8% water glass (modulus 1.0). This optimum aligns with the fundamental principles of balancing activator alkalinity and silicate modulus in alkali-activated system design.
(2) Hydration Process Characteristics Revealed by Thermal Analysis. The thermal decomposition behavior provided critical thermodynamic insights. The anomalous weight gain observed at 3 days indicates an active secondary hydration reaction, attributable to the continuous alkaline dissolution of silico-aluminous phases and subsequent gel formation. By 7 days, the primary hydration is substantially complete, coinciding with a densification of the microstructure. This sequence confirms the high activation efficiency and progressive reaction nature of the composite activator.
(3) Kinetic Mechanism Elucidated by the Krstulović–Dabić (KD) Model. The hydration kinetics of the alkali-activated CWM system were successfully quantified using the KD model. The process is best described as a sequential evolution through three controlling stages: nucleation and growth (NG), phase boundary reaction (I), and diffusion (D). Specifically, the early rapid reaction (before ~0.15 h) is dominated by the NG process, which then transitions to the I process (0.21–0.5 h). The subsequent deceleration (20–50 h) is governed by a combination of I and D processes, before the reaction enters a stable period beyond 50 h where diffusion (D) becomes the sole rate-limiting step. This model quantitatively yielded the reaction rate constants (*K*_NG_, *K*_I_, *K*_D_), the Avrami exponent (n), and the stage transition points *(α*1, *α*2), providing a mechanistic explanation for the “early-strength and rapid-hardening” characteristics observed in the composite alkali-activated system at a fundamental kinetic level.

Collectively, these findings establish a material design framework guided by activator optimization, substantiated by thermodynamic evidence, and explained by a robust kinetic model. The work demonstrates that the KD model is a powerful tool for deciphering the complex hydration behavior of alkali-activated CWM, offering theoretical guidance for the development of performance-controllable sustainable cementitious materials.

### 6.2. Future Research Directions

While this study provides fundamental insights into the hydration kinetics and activation mechanisms of alkali-activated CWM, it represents an interim step within a broader research landscape. Several critical and specific directions emerge for future exploration, directly addressing the limitations of the present work and aiming to bridge the gap between laboratory understanding and practical application.

(1) Diversification and Mechanistic Optimization of Activation Systems.

The current study compared a limited set of activators. To achieve performance-tunable binders, future work must expand the activator portfolio. This includes investigating alternative alkaline sources (e.g., KOH, Na_2_CO_3_) and multi-component systems (e.g., blends of sodium silicate with different moduli and NaOH). Crucially, this exploration should be mechanism-driven. Building upon the established Krstulović–Dabić (KD) kinetic framework, research should quantify how these different activators specifically alter the stage-specific rate constants (*K*_NG_, *K*_I_, *K*_D_), transition points (*α*1, *α*2), and the resulting microstructure. The goal is to move beyond empirical mixing towards a predictive model that links activator chemistry to reaction kinetics and final gel properties.

(2) Long-Term Engineering Performance and Durability under Realistic Conditions.

For engineering adoption, the long-term behavior of alkali-activated CWM must be addressed. Key challenges include high drying shrinkage, alkali effusion, and workability control. Future research needs to develop targeted strategies:

Shrinkage Mitigation: Explore the efficacy of specific shrinkage-reducing admixtures, internal curing agents, or optimized aggregate grading, with the goal of achieving a drying shrinkage strain below a benchmark value for structural applications.
Durability in Aggressive Environments: Conduct long-term exposure studies to evaluate performance under combined sulfate, acid, and chloride attacks. A promising approach is to correlate the diffusion-stage rate constant (*K*_D_) from the KD model with the long-term transport properties and degradation resistance, establishing a kinetics-based durability indicator.
Rheology and Constructability: Systematically study the influence of CWM fineness, activator type, and admixtures on rheological properties to meet the demands of diverse casting processes like pumping or 3D printing.
(3) Holistic Sustainability Assessment via Life Cycle Analysis (LCA).

To fully substantiate the environmental promise of alkali-activated CWM, a comprehensive life cycle assessment (LCA) is essential. This study focused on the material’s intrinsic reactivity. The next step is to quantify the full carbon and energy footprint—from CWM sourcing and processing, through activator production, to final application and end-of-life scenarios. This LCA should provide a clear, quantitative comparison with ordinary Portland cement, explicitly demonstrating the contribution of this technology to the “dual-carbon” goals in the construction sector. Such data is critical for informing policy, securing funding, and enhancing market competitiveness.

## Figures and Tables

**Table 1:** Chemical composition of CWM and cement (wt%).

Compositions	CaO	SiO_2_	Al_2_O_3_	Fe_2_O_3_	MgO	SO_3_	K_2_O + Na_2_O	Loss
CWM	12–14	60–65	10–15	3–8	1–1.8	0.8–1.0	0.8–3.2	2–4
Cement	63.81	21.60	4.35	2.95	1.76	2.06	0.67	2.80

**Table 2:** Specific surface area and different particles sizes content of CWM particles.

Particle Characteristics	Specific Surface Area/m^2^·kg^−1^	<1 μm %	1–3 μm %	3–10 μm %	10–20 μm %	20–30 μm %	30–40 μm %	>40 μm %
CWM	1595	5.8	20.99	38.45	18.94	8.78	3.91	3.13

**Table 3:** Mineral composition of CWM (%).

Compositions	SiO_2_	CaCO_3_	Kaolinite	Illite	Dolomite	Feldspar
CWM	48–62	20–34	1–5	5–10	5–10	15–20

**Table 4:** Mix proportions of alkali-activated CWM system composite paste.

Sample	Single Activator			Sample	Two-Component Alkali			
	P.O.	CWM	Water		P.O.	CH	CWM	Water
S0	450	0	129	S10	270	24	180	171
S1	360	90	137	S11	270	48	180	175
S2	270	180	147	S12	270	72	180	178
S3	225	225	151	**Sample**	**P.O.**	**WG (Module)**	**CWM**	**Water**
**Sample**	**CH**	**CWM**	**Water**	S13	270	14 (1.0)	180	139
S4	54	450	178	S14	270	18 (1.0)	180	139
S5	36	450	175	S15	270	27 (1.0)	180	139
S6	18	450	171	S16	270	14 (2.3)	180	160
**Sample**	**WG (Module)**	**CWM**	**Water**	S17	270	18 (2.3)	180	160
S7	67.5 (1.0)	450	139	S18	270	27 (2.3)	180	160
S8	67.5 (2.3)	450	160	S19	270	14 (3.3)	180	158
S9	67.5 (3.3)	450	158	S20	270	18 (3.3)	180	158
				S21	270	27 (3.3)	180	158

Note: ① The mix proportions are given by mass g. The water content was adjusted to achieve the standard consistency of the paste according to GB/T 1346 [18]. The estimated binder content per cubic meter is calculated based on the density of the fresh paste. ② The binding materials are CWM and P.O., with their masses being 500 g each and remaining constant. CH and WG are the alkaline activators, and they are the additional amounts added to the total amount of the cementitious materials.

**Table 5:** Mass loss percentages at key temperature intervals for the dual-alkali activated CWM paste at 3 and 7 days.

Hydration Products	Decomposition Temperature Range (°C)	Mass Loss at 3 Days (%)	Mass Loss at 7 Days (%)	Change (7 d–3 d)
free water	<110 °C	−9.03	−6.00	+3.03
AFt	100~120 °C	−0.80	−0.21	+0.59
C-S-H gel	120~388 °C	−6.10	−5.00	+1.10
Anomalous Region	388~465 °C	+3.28	−1.02	−4.30
Ca(OH)_2_	465~574 °C	−3.89	−1.60	+2.29
High-Temperature Region	574~637 °C	+0.92	−1.47	−2.39
CaCO_3_	>637 °C	−10.40	−6.35	+4.05
Total Mass Loss	/	−26.02	−21.65	+4.37

**Table 6:** Comparison of the KD model with other common hydration kinetic models.

Model Name	Core Idea	Key Features	Main Limitations
JMAK [49,50,51,52,53]	Hydration is considered a phase transformation process where stable nuclei form randomly in the liquid phase and grow three-dimensionally.	The Avrami equation is used to describe the change in hydration degree over time, assuming random nucleation and constant growth rate.	This model lacks physical significance and cannot describe the mechanism transformation.
Boundary Response Model [54]	The reaction rate is controlled by the movement of the phase interface.	Applicable to the description of mid-stage hydration reactions.	An idealized dynamic description. In reality, cement hydration is a complex phenomenon involving multiple interwoven processes.
Diffusion-Controlled Model [55]	The growth space of the hydration products is limited by physical or chemical factors rather than unlimited, which directly leads to the transition of the hydration reaction from the acceleration phase to the deceleration phase.	Applicable to the description of the transition from the acceleration phase to the deceleration phase.	For reactions with complex mechanisms, sometimes simple limited growth models fail to capture the entire process accurately.
KD Model [56]	The complex cement hydration process is decomposed into a dynamic framework, which consists of three basic control stages: NG, I, and D.	Comprehensively describe the changes in the hydration process of composite cementitious materials.	Many parameters require precise experimental data.

**Table 7:** Hydrolysis kinetic parameters of CWM activated by alkali.

n	*K*_NG_	*K*_I_	*K*_D_	*α*_1_	*α*_2_
1.52	0.0244	0.0946	0.00753	0.016	0.433

**Table 8:** Comparison of hydration kinetic parameters of alkali-activated CWM with other cementitious materials.

System	n	*K*_NG_	*K*_I_	*K*_D_	*α*1	*α*2
Ordinary Portland Cement [59,60]	1.63–1.92	0.0537–0.0578	0.012–0.016	0.0025–0.0034	0.145–0.267	0.232–0.332
Slag + Cement [59,61]	1.42–1.88	0.028–0.177	0.005–0.018	0.0017–0.0080	0.070–0.195	0.180–0.311
Brick Powder + Cement [62]	1.71–1.73	0.0382–0.0385	0.084–0.0085	0.0014–0.0015	0.111–0.119	0.224–0.227
Alkali-Activated Slag [27,63]	1.40–1.70	0.017–0.035	0.0025–0.0060	0.0003–0.0014	0.149–0.253	0.218–0.346
Alkali-Activated CWM	1.52	0.0244	0.0946	0.00753	0.016	0.433

## Data Availability

The original contributions presented in this study are included in the article. Further inquiries can be directed to the corresponding author.

## Abbreviations

The following abbreviations are used in this manuscript:

CWM	Construction waste micro-powder
P.O.	Portland cement
CH	Ca(OH)_2_
WG	Water glass
